# Contextual factors and sporting success: The relationship between birth date and place of early development on the progression of Jamaican track and field athletes from junior to senior level

**DOI:** 10.1371/journal.pone.0227144

**Published:** 2019-12-27

**Authors:** Eon Campbell, Rachael Irving, Melanie Poudevigne, Lowell Dilworth, Shelly McFarlane, Olusegun Ismail, Janel Bailey

**Affiliations:** 1 Department of Basic Medical Sciences, The University of the West Indies, Mona, Jamaica; 2 Health and Fitness Management, Clayton State University, Morrow, Georgia, United States of America; 3 Department of Pathology, The University of the West Indies, Mona, Jamaica; 4 Caribbean Institute for Health Research, The University of the West Indies, Mona, Jamaica; 5 Department of Mathematics & Statistics, The University of Technology, Kingston, Jamaica; Universidade Federal de Mato Grosso do Sul, BRAZIL

## Abstract

Understanding determinants associated with dropout from sport is important for talent development. This study aimed (i) to determine dropout rates for Jamaican track and field athletes and (ii) to examine contextual factors (i.e., relative age effect and place of development) as potential determinants of junior athletes progressing to the senior level. A sample of 1552 track and field athletes (mean age 18.57±0.41 years) who were finalists at the national high school (junior) championships in Jamaica between 2000 and 2017 were evaluated from the Jamaica Athletics Administrative Association database. The database provided birth date, school attendance and performance results. A retrospective analysis was completed to investigate the relationship between junior and senior successes and dropout rates. Chi-square analyses were conducted to examine the distribution of birth date quartiles based on the selection year. Using the Jamaican census information, the population size of regions where participants attended school were categorized and used as a proxy for athletes’ place of development. Results showed that the majority of the participants did not progress to senior levels (81%). The relative age effect was evident for athletes who progressed to the senior level but was not evident for athletes who did not progress. There was a bias towards participants who attended school in regions with a population size between 5000–29 999. This study illuminates some of the contextual factors that may influence the likelihood of progressing from junior to senior levels which may help to inform talent identification, selection and development in the sport of track and field.

## Introduction

With the ensuing benefits of sports worldwide, there is a growing interest by nations to perpetuate long-term and sustainable talent development in sport performance. As a result, governments have invested vast resources in the pursuit of sporting success [1–2]. Sporting success can be measured by the number of athletes reaching the podium consistently at international sporting events [2]. Nations have invested significantly in financial and human resources to attain sporting success. Nevertheless, numerous studies in certain nations are highlighting an increase in sports dropout in children aged 10–20 years [3–5]. Consistent reports in the literature show that, by the time children reach the age of 15 years, 70–80% no longer participate in sports. For example, a longitudinal study over the span of 25 years with Norwegian track and field athletes highlighted up to 75% of athletes ages 13 to 16 years drop out along the way [4]. Studies focusing on the progression from the junior to the senior levels in sports have illuminated only a few junior champions, approximately 9% of males and 12% of females track and field athletes in the United Kingdom [6] and a staggering 17% of all sports participants [7], progressed to the senior level. The precise mechanism(s) for sport dropout are unclear. Some likely reasons include: overtraining, injuries, lack of motivation and alternative leisure-time activities [4].

To improve progression from elite junior to elite senior levels of sport, a clear understanding of talent development is necessary. There has been growing interest in talent research in the past decade [8–11]. A significant portion of this research has been devoted to examining the quality and quantity of meaningful practice, also known as deliberate practice [12]. These studies [13–14] illuminated that athletes who begin goal-oriented and systemic training at an early age has a greater likelihood for performance advantages. The authors contend that the lead in performance is distinct between individuals of the same calendar year who started training at a different age. At the same time, research has shown the disproportion in the number of “successful” athletes born within the first three months of the selection year in comparison to athletes born at the end of the year. This phenomenon is termed: relative age effect (RAE) [15]. One dominant explanation for this phenomenon relates to the ‘maturation-selection hypothesis’ [15–17]. According to this hypothesis, athletes who are older and have more time to mature physically and mentally are often selected, compared to their younger counterparts [18]. Thus, the relatively older and earlier-maturing athletes are more likely to be considered for selection [17,19]. Unfortunately, the relatively younger and later maturing athletes are more likely to be overlooked and excluded. Helsen and colleagues’ [20] study on the influence of the RAE on Belgian soccer players achievement showed early born youth-level athletes are more likely to be identified as talented and be exposed to higher levels of coaching than their later-born counterparts. This in turn may play a role in drop-out rates for the relatively younger athletes [20–21].

The RAE has been widely studied in sports science revealing consistent prevalence with physical demand, level of practice, age and gender acting as moderating variables [15–17]. Generally, physically demanding sports (e.g., tennis and swimming) are more affected by the RAE in contrast to sports with emphasis on skills (e.g., golf) [7, 16, 17]. When the RAE is found to be present, it tends to be apparent through most age groups, with weaker effects in adulthood [22]. Studies have found some differences in the RAE according to the level of practice. For example, in soccer, Práxedes and colleagues [23] observed the higher the level of training, the larger the RAE, however, the observed pattern may have been due to inter-relations between deliberate practice and the RAE [24]. To date, there is limited evidence of the RAE in track and field [16, 25–26]. In one of the few studies, Brazo-Sayavera and colleagues [16, 25] highlighted the influential role of RAEs on selection opportunities in track and field athletes. The authors showed that age and gender were mediators with a stronger effect on males and younger athletes. In another example, Romann and colleague [17] investigated sprint performance and the RAE in youth contexts and found that while the RAE was evident throughout the age groups, it was mediated by age and performance. The authors found the RAE had significant implication in the selection of sprinting athletes during adolescence. Female representation, however, was not available in the sample investigated by Romann and colleague [17], which highlights a disparity in the literature in RAEs research and in the greater context of talent research as well [27]. The majority of studies have focused on RAEs throughout the period of adolescence or in adulthood [15–17]. In contrast, there is a dearth of studies investigating the influence of the RAE in determining the likelihood of a junior athlete progressing to the senior level in sport. In their work, Wrang and colleagues [28] found that the youth level favored relatively older athletes, of whom a large proportion failed to be re-selected as a senior compared to their younger counterparts.

It should be noted that some studies have observed different distributions of the birthdate of athletes, where athletes born at the end of the selection year earn more than their teammates born at the start of the year [29]. It is possible other factors could be moderating the effect. For example, evidence highlights the crucial role an individual’s early sporting environment can have in relation to their sport success. In some cases [30–32] the size of the community where an athlete spent his/her early stage of development can affect the likelihood of attaining elite performance. This phenomenon has been demonstrated in both professional and amateur levels, in sports such as ice hockey [30], soccer [31] and swimming [32]. Generally, findings revealed ‘small’ to ‘medium’ community sizes (between 1000–499 999 inhabitants) tend to have/produce a higher proportion of elite athletes compared to communities larger than 500 000 inhabitants. For example, Allen and Duncan [33] examined elite athletes from the United Kingdom (UK) and highlighted that athletes in the World Class Program were 2.1 times more likely to be born in a ‘medium-sized’ community compared to the general population in the UK. A variety of explanations have been proposed to account for this phenomenon, which includes better psychosocial support and appropriate physical environments in small communities [34–36]. It has been argued that adolescents in small communities receive more social support, have higher levels of self-efficacy, and experience fewer conflicts [36]. Additionally, small communities may provide a safer environment with increased recreational space, which may be more conducive to an athlete’s development [37–38].

Previous studies examining the influence of place of development (POD) on sporting success used the population of the athlete’s birthplace as a proxy where formative sport developments took place [30–31, 34, 39–40]. While the place of birth may be an important consideration, there is contradictory evidence to show otherwise [35]. Researchers have discussed that the birthplace is not necessarily the POD. For example, individuals born in small rural areas might move to larger urban centers during their childhood or vice versa. Due to the possibility of incongruence between locations, studies [35, 41] have proposed that the location where an athlete first attain training experience may be a more appropriate measure of an athlete’s early development context. In Western societies, most athletes acquired sporting skills within the school environment. On average, an athlete may spend as much as 10 hours each day at school. This means that the school environment may play a great role in an athlete’s development. Accordingly, Allen and colleague [33] found that elite athletes were 10.5 times more likely to attend a primary school in small communities and 3 times more likely to attend secondary schools in small communities. For these reasons, birthplace may not be as critical as the place of early development [41]. Thus, it has been proposed the size of the community in which an athlete attended school may be a more appropriate measure of the developmental context that influences progression in sports.

In Jamaica track and field is a popular individual sport among adolescents. Over 3000 adolescents who are enrolled in high schools annually across Jamaica, compete in track and field events at the Inter-Secondary School Sports Association Championships (ISSA CHAMPS) [42]. ISSA CHAMPS is Jamaica’s largest junior track and field development program. It aims to foster talent development through early specialization and increased training during the early stages of development in junior athletes. An analysis of the progression rate from the junior to the senior levels may provide evidence for the effectiveness of the development program. There is anecdotal evidence for Jamaican propensity for speed. However, only a few scientific explanations have explained Jamaican dominance in the sprints. Three main avenues for understanding the superior Jamaican performance in athletic events have emerged: genes [43], physiology [44] and the socio-cultural environment [45]. In the general sports science literature, sporting success has been addressed from a wide perspective focusing on many levels of determinants. Particularly the performers’ characteristics such as birthdate, genetics, physiology and psychology, the environment (birthplace and support programs) and training programs [10–11]. Surprisingly, there is a lack of current knowledge with regards to the effects of most of these determinants in Jamaica’s sporting success. Therefore, the goals of this article are: (1) to determine the drop out and progression rates of Jamaican junior track and field athletes, and (2) to investigate whether contextual factors such as birth dates and POD would influence the likelihood of progression from junior to senior levels.

## Materials and methods

### Participants

Data for 1552 athletes (54% male and 46% female) who made the finals (top 8) of individual events at the ISSA CHAMPS between 2000–2017 were extracted from the Jamaica Athletics Administrative Association (JAAA) database. All athletes at the time of their participation at ISSA CHAMPS were age 18 and 19 years (mean age 18.57±0.41 years). The mean age of the females was 18.70±0.31 years while the mean age of the male was 18.43±0.50. The athletes participated in the sprint/hurdle (44%), middle/long distance (21%) and jump/throw (36%) events. Participants who competed in two or more competitive events were recorded as an individual athlete and their best overall performance in an individual event was used for the analysis. For those who competed during numerous seasons, the initial year he/she represented the country at the senior level was used for analyses. This study was approved by the University of the West Indies Ethics Committee (Mona) and is in accordance with the Declaration of Helsinki. Informed consent was not needed as the study analyzed and reported data that is available to the public domain.

### Procedure

#### Progression analysis

A retrospective analysis was done to investigate the relationship between junior and senior successes. Data were acquired from the publicly-available JAAA database. Performance results, school attended, date of birth, age, sex and athletic event were extracted from the database. The identities of the participants were matched against the identities of athletes placed in the finals (top 8) at Jamaica’s National Senior Championships. Participants mutually identified as ISSA CHAMPS and National Senior Championships finalists were categorized as “progressed”. The identities of finalists at ISSA CHAMPS were also compared to the identities of Jamaican representatives at the Commonwealth Games, the senior editions of International Association of Athletics Federation (IAAF) World Championship Games and the Olympic Games. Participants who were not identified among the national senior finalists or the international representatives, either did not compete or did not rank high enough, were categorized as “did not progress”. Finally, the years the athletes took for progression from junior to senior levels of competition was determined by subtracting the last year the athlete was identified as an ISSA CHAMPS finalist (prior to becoming a national senior finalist), from the first year the athlete competed at Jamaica’s National Senior Championships.

#### Relative age effect

Birth month of each athlete was categorized into quarters (Q), reflective of the calendar year of the junior track and field season. The calendar year of Jamaica’s junior track and field is from the 1^st^ of September to the 31^st^ of August [42] (Q1 = born in September–November; Q2 = December–February; Q3 = March–May; and, Q4 = June–August).

#### Place of early development

The high schools the athletes attended were recorded then collapsed into region sizes determined by the 2011 Population and Housing Census for Jamaica [46]. Region sizes were eight categories based on population: category 1 (> 500 000), category 2 (499 999–250 000), category 3 (249 999–100 000), category 4 (99 999–30 000) category 5 (29 999–10 000) category 6 (9999–5000), category 7 (4999–2500) and category 8 (< 2500). Since the examination includes athletes who were ages 18 and 19 years, the 2011 Census Data for Jamaican Youths (age 14 – 24years) [46] was used to calculate the expected percentage of athletes in each category. This census year is assumed to be an accurate representation of the athletes under investigation. It is important to note the high schools the athletes attended were used to provide a proxy for the location in which junior athletes spent their developmental years. It is also important to recognize the school an athlete attended may not always coincide with the POD. Although migration is probable within our sample, net movements are likely to be essentially equal [46].

### Statistical analyses

Chi-square tests were applied to analyze the proportion of junior finalists and the proportion of athletes who were national senior finalists and international senior representatives. This statistical procedure has previously been applied in the retrospective analysis of progression in track and field athletes [6]. Chi-squared tests were also performed on the birth date of each athlete, categorized as quartiles of the track and field calendar year to determine the significance of deviations for the expected number of births in each quarter [30, 39]. Equal distribution of births was assumed for each quarter of the selection year [17, 28]. Further chi-square analyses were done to determine the associations between relative age and gender and athletic event. In this study, Cramer’s V provided the magnitude of the effect size where 0.06 < V ≤ 0.17 indicated a small effect, 0.17 < V < 0.29 a medium effect, and, V ≥ 0.29 a large effect.

In determining the POD effect on the progression from juniors to senior athletic representation, odds ratios (ORs) were calculated across different region sizes within a 95% confidence interval (CI) [35, 37, 39]. An OR greater than 1 (i.e., upper and lower CIs greater than 1) implied that an athlete who attended school in a region of a given size (e.g., in a region with 9999–5000 inhabitants) was more likely to become a senior representative than if the athlete attended school in a region of any other size. An OR less than 1 (i.e., upper and lower CIs less than 1) implied that an athlete who attended school in a region of a given size (e.g. in a region with ≥ 500 000 inhabitants) was less likely to become a senior representative than if attended school in a region of any other size. An OR with a CI range that included the null value of 1 was not considered significant.

Multiple additional analyses were conducted to determine the associations between the RAE, POD and years taken for progression from junior to senior levels of competition. First, Spearman rank correlation and chi-square analysis were conducted to determine the relationships between birth month quarters and region sizes (based on the location of school attendance). Second, the relationship between years taken to progress and birthdate and region size were examined using Spearman rank correlations and chi-square. This was necessary to examine if relative age and POD were related in any way to the years the athlete took for progression from junior to senior levels of competition.

## Results

### Progression analysis

**Fig 1:**
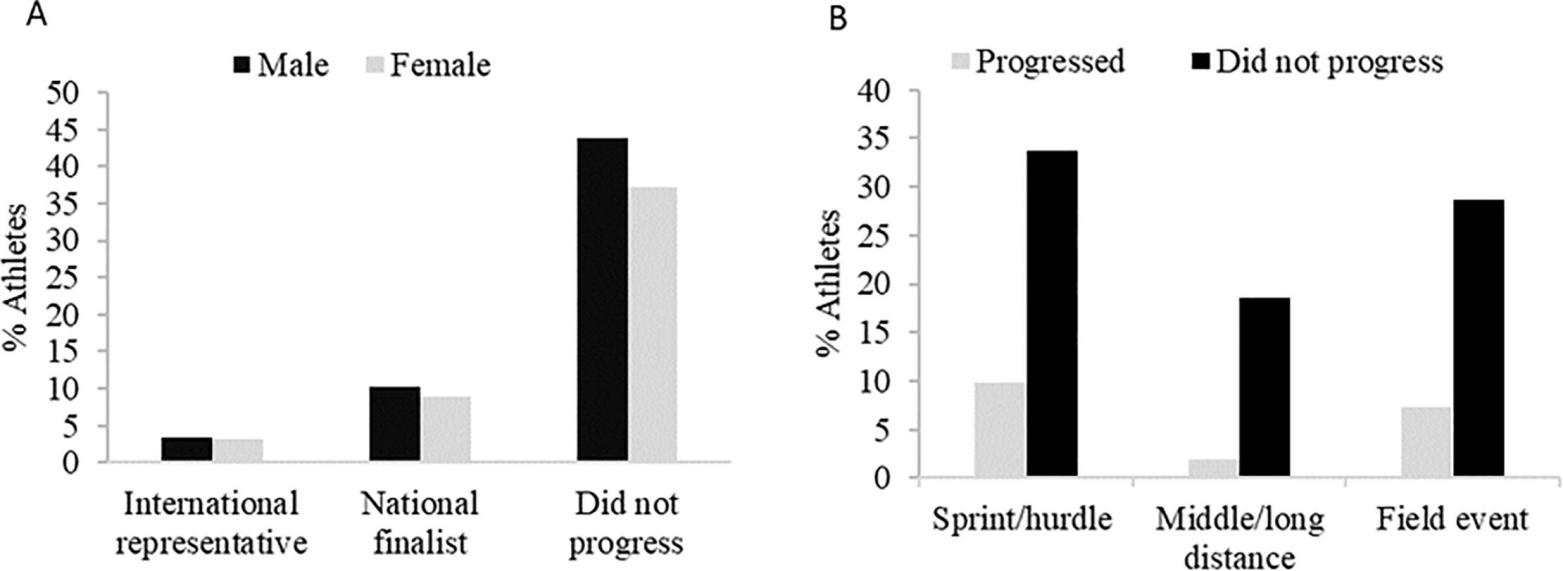
The percentage of junior finalist by genders (A) and by athletic events (B) who progressed and who did not progress to senior levels of competition.

Fig 1A illustrates the percentage of Jamaican junior finalists who progressed (19%) to senior levels of competition and those who did not progress (81%). In both genders, the majority of the participants did not progress to the senior levels (43.9% of males vs. 37.1% of females). Approximately, 10.2% of the male and 8.8% of the female athletes progressed to the senior levels. With regard to international representation, only a small portion of the athletes (3.4% of males vs. 3.1% of females) represented Jamaica at the international levels. The proportion of male and female athletes did not differ among the national senior finalists (χ2 (1) = 0.04, V = 0.005, p = 0.85) or among the international senior representatives (χ2 (1) = 0.11, V = 0.008, p = 0.74). There were significant differences in the athletic events of the progressed athletes (χ2 (2) = 5.15, V = 0.13, p = 0.02). An over-representation was observed in the sprint/hurdle athletes while there was an under-representation in the middle/long distance athletes (Fig 1B). The years athletes took to progress from junior to senior levels of competition ranged from 0 years (meaning the athlete was identified as a junior finalist and as a senior finalist in the same year) to 12 years.

### Relative age effect

Table 1 illustrates the frequency and percentage distribution of athletes’ birth month and the results of the chi-square analyses. The analyses did not identify the RAE for all junior finalists (age 18–19 years) and for athletes who did not progress to senior levels of competition. However, the results identify the RAE for the athletes who progressed to senior levels of competition, showing a large and significant effect size (V = 0.40). There was an over-representation in the progressed athletes who were born in the first quarter of the athletic year compared to athletes born in the last quarter of the athletic year. No main effect of gender (χ2 = 0.10, V = 0.01, p = 0.10) or athletic event (χ2 = 9.43, V = 0.06, p = 0.15) was observed for the RAE.

**Table 1:** Chi-square values and related probabilities between observed frequencies and expected frequencies.

	Frequency and % of athletes per quarter							
Athlete	Q1 (%)	Q2 (%)	Q3 (%)	Q4 (%)	Total	χ2	*V*	*P*
All	401 (25.8)	373 (24.0)	383 (24.7)	395 (25.4)	1552	1.21	0.06	0.85
Did not progress	304 (24.2)	300 (23.9)	314 (25.0)	339 (26.9)	1257	2.93	0.10	0.40
Progressed	97 (32.9)	73 (24.7)	69 (23.4)	56 (19.0)	295	11.92	0.40	0.001*

Significant at p < 0.05*

Abbreviation: Q–quarter

### Place of development effect

Table 2 provides data from the 2011 Population and Housing Census on the percentage of youths (age 14–24 years) who lived in regions of different sizes and the percentage of junior track and field finalists (age 18–19 years) who attended schools in these different regions, between the years 2000 and 2017. The table also contains the odds ratios of a junior finalist becoming a senior representative by the attendance of schools in regions of varying sizes in Jamaica. Regions with a population between 5000 and 29 999 present the best odds of producing Jamaican senior track and field representatives. The results showed that the junior track and field finalists from regions with population 5000–29 999 were 1.8 to 1.9 times more likely to become a Jamaican senior representative.

**Table 2:** A representation of Jamaica general youth population and the population of Jamaican junior track and field athletes with odds of them becoming a senior representative based on the size of the region for the athlete’s school attendance.

Region size	% General population ^a^	% Athletes ^b^	OR (95%CI)
> 500 000	40.4	38.8	0.94 (0.73, 1.23)
499 999–250, 000	0	0	-
249 999–100 000	30.4	15.5	0.80 (0.79, 1.61)
99 999–30 000	7.7	4.6	0.78 (0.90, 1.82)
29 999–10 000	10.5	17.3	1.89 (1.06, 3.45)
9999–5000	5.6	12.2	1.80 (1.04, 3.06)
4999–2500	4.3	10.7	1.19 (0.82, 2.06)
< 2500	1.0	0.9	0.98 (0.61, 1.56)

^a^ Percentage of youth (age 14–24 years) in each subdivision of the 2011 Jamaican census.

^b^ Percentage of ISSA CHAMPS finalists between 2000 and 2017 in each subdivision of Jamaica.

Abbreviations: OR- odds ratio, CI—confidence interval

### Interaction between birth date and place of development

The possible interaction between birth date (according to the sport calendar year) and POD (according to the school attended in different region sizes), was examined using chi-square analysis and Spearman rho correlation. Results of the analyses were not significant: chi-square (χ2 (18) = 18.23, p > 0.05) and Spearman rho correlation (r = 0.06, p > 0.05). These results showed that birth date and POD appear to be independent of each other. Analysis for interactions between years taken for progression with birth date and POD indicated the years athletes took for progression from junior to senior levels of competition were not associated with either birth date (χ2 (9) = 1.72, p > 0.05) or POD (χ2 (18) = 12.47, p > 0.05).

## Discussion

This paper adds to the existing literature on the progression of athletes from elite junior to senior levels of competition. Uniquely, the study examined the extent to which factors such as birth date (RAE) and POD are associated with the progression of Jamaican junior finalists to elite senior levels of competition. The results indicated that 81% of Jamaican junior finalists did not progress to become national senior finalists. This finding is consistent with studies in a range of sports [3, 7] including track and field [4, 6]. The results also revealed there are significant differences in the athletic events of the Jamaican junior athletes who progressed to senior levels. There is a significant over-representation of sprint/hurdle athletes whereas there is an under-representation of middle/long-distance athletes. It is not clear if this finding is due to the greater emphasis being placed on the sprints in Jamaica or if there is a variation in the peak age of distance running compared to sprinting. Nevertheless, research has attributed the Jamaican propensity for speed to the socio-cultural environment, as well as to genetics and physiology [43–45]. These attributes alone are not sufficient to understand the dropout rate from youth sports. Further research is needed to investigate psychosocial factors (such as intra–inter–personal factors) to help understand the potential determinants of Jamaican youth sport dropout for each athletic event. Moreover, the finding that the majority of Jamaican junior finalists did not progress to senior levels reinforces that excelling as a junior is not necessarily a prerequisite for success at the senior levels [7].

The results of the study partially support the RAE in Jamaican athletes. The RAE was not consistently observed in the 18–19 years old track and field athletes and athletes who did not progress to senior levels. However, the RAE was evident in the athletes who progressed to senior levels of competition. Specifically, for those who progressed to senior levels of competition the data showed a significant over-representation of athletes born in the first quarter (Q1) of the athletic year and a significant under-representation in the number of athletes born in the last quarter (Q4) of the athletic season. This finding showed a similar pattern in RAEs of the works carried out in track and field by Brazo-Sayavera and colleagues [16]. In the current study, the RAE effect size was large for athletes who progressed to senior levels in comparison to the small, non-significant effect size seen for the athletes who did not progress. The finding in the current study is well-aligned with the evidence of increasing RAEs with performance level [16–17]. Our finding contradicts a recent study by Wrang and colleagues [28] who showed significant RAEs at both youth and senior national levels. The absence of RAEs at the youth level and the appearance of RAEs in athletes with successes at both junior and senior levels of competition could be due to the relatively older athletes starting sports first (i.e., training and competing at an earlier age) thus having earlier sport experience. The earlier sport experience may lead to higher accumulated training volume which Ericsson and colleagues [12] contend may be advantageous to performance at every stage of life.

The high school the athlete attended was used as a proxy to determine how the athlete’s development environment affected the likelihood of achieving an elite performance level. From a theoretical perspective, studies posited that contextual factors such as psychosocial climate and physical environment vary with population size and provide athletes with different sporting opportunities. In the current study we reinforced that an athlete’s POD may affect the likelihood of achieving elite performance [30, 37, 39]. We found that athletes who attended a high school in regions with 2500 to 29 999 inhabitants (areas classified by the 2011 Jamaica Population and Housing Census as urban centers) were systematically overrepresented at the senior levels, in comparison to athletes with school attendance in regions > 29 999 inhabitants. Research presented findings that cities with small population sizes (e.g., the urban centers in Jamaica) have better psychosocial and physical environments than cities with large population sizes [34]. Some possible benefits that have been proposed to be present in smaller cities include: (i) increased access to spaces supporting deliberate play and practice [30] and (ii) coherent development and support systems which emphasize appropriate development rather than early success [47]. These benefits are however, largely speculative in nature and have not been comprehensively examined [35, 47]. Additionally, it has been shown that individuals from small cities may receive more social support and have higher self-efficacy [36] in part due to the closer intimacy provided by the small city setting. Generally, most small cities tend to have appropriate infrastructures such as sporting facilities and coaches, as well as large green spaces [30, 35, 37]. Public green spaces can provide youth with more space for deliberate play and unorganized physical activity, factors which are associated with sport participation and sporting success [13, 48]. Smaller populated cities have been recognized as being safer, less structured and more spacious [37–38]. These characteristics are believed to be associated with continued involvement in sports worldwide [48]. Interpreting the results of these studies with the interactions of the psychosocial climate, the green spaces and the infrastructural benefits offered by small cities, could be the reason why we found that schools in regions with 5000 to 29 999 inhabitants were 1.8–1.9 times more likely to produce athletes that will progress to the senior levels compared to athletes from larger cities.

The current study supports previous findings showing that large cities and very small communities may present a disadvantage in the development of elite athletes [30, 37]. We found that athletes who attended schools in regions with more than 30 000 inhabitants (classified by the 2011 Jamaican Population and Housing Census as a large capital town) had disproportionately fewer athletes. Similarly, regions with less than 2500 inhabitants (classified by the 2011 Jamaican Census as small rural communities) had disproportionately fewer athletes moving from the junior to the senior levels of competition. Although large cities are likely to have appropriate infrastructures, they may also have fewer access to space supporting deliberate play [31]. Larger cities also offer a greater variety of alternative activities, such as music and arts which are consequently cited as motives for sports withdrawal [49]. On the other hand, smaller rural communities may lack adequate infrastructure for talent development. The notion that the smaller and more densely populated regions are less effective in producing elite athletes complement previous work by Côté and colleagues’ [30] and Hancock and colleagues’ work [37] who used birthplace as a proxy for athletes’ developmental environment. These authors found that the odds of attaining elite status significantly decreased for athletes who practice sports in very large or very small cities.

This was the first study to examine the possible interaction between birth date, POD, and years taken for progression from the junior to the senior levels of competition. The inter-relations between birth date and POD revealed no significant interaction, which supported the results of previous studies [30, 35]. These findings add to existing literature that RAEs may not moderate the POD effect in predicting youth progression in sports. Baker and colleague [39] found that relative age and birthplace size effects were related to the players’ selection round in hockey leagues in Canada. From their findings, we hypothesized that birth date and POD are related to years taken for progression from junior to senior levels of competition. However, our findings did not support the hypothesis that birth date and POD were related to years taken for progression, despite both the RAE and POD having some impact on the junior to senior progression in Jamaican track and field athletes.

## Limitations

Notwithstanding, the present study has limitations which should be addressed. Firstly, the study was retrospective and provided statistical data reveling only some of the factors contributing to the dropout problem. Second, the database did not contain historical data such as the athlete’s history of injuries and whether the athlete wanted to become a pro-athlete or not. As such, when athletes were identified as a junior finalist, but not as a senior, it is not clear whether the athletes completely dropped out of sport, were participating in other sports or were just not performing well enough to make the national team. Finally, like other birthplace-effect studies which used population categories [29, 36, 38]. The use of population categories in this study as a proxy for POD is limited, as there might be sizable variation within the population density of each district. Future research may seek to use alternative methods to limit such variability, thus enabling stronger within-category consistency.

## Conclusions

The results of the study highlight that the majority of Jamaican junior track and field athletes did not progress to the senior levels. In an attempt to understand dropout rates from the sport, we examined the impact of RAEs and the effect of POD on the progression of Jamaican track and field athletes from the junior to senior levels of competition. The results showed that both birthdate (RAE) and POD were important determinants in the junior to senior athletic progression. We observed that the RAE was not evident in the athletes who did not progress but was evident in the athletes who progressed to senior levels of competition. Drawing from the published existing evidence, the Ericsson and coworkers’ [12] theory of deliberate practice was acknowledged as a possible factor that contributed to the observed phenomenon. Additionally, it was found that sport environments in small cities (urban centers from a Jamaican perspective) presented the best odds of producing juniors who progressed to senior levels. It has been theorized that small cities may have a better physical environment and psychosocial climate, for talent development. Birth date and POD were also found to be independent predictors of junior to senior progression in track and field. Overall this study provided preliminary evidence of factors associated with dropping out from sports between the junior and the senior levels. This study adds to the growing body of literature suggesting that contextual factors are important determinants for sports dropouts. However, the study was retrospective and only provided statistical data revealing some of the factors involved in the dropout problem but not covering all factors. Qualitative research is needed to gain a clearer understanding of the exact contextual factors which influence the phenomenon of sports dropouts.

## References

[1] Huxley DJ, O’Connor D, Larkin P. The pathway to the top: Key factors and influences in the development of Australian Olympic and World Championship Track and Field athletes. Int J Sports Sci Coa. 2017 2 21;12(2):264–275.

[2] Sotiriadou KP, Shilbury D. Australian elite athlete development: An organizational perspective. Sport Manag Rev. 2009;12(3):137–148.

[3] Barreiros A, Côté J, Fonseca AM. From early to adult sport success: Analyzing athletes' progression in national squads. Eur J Sport Sci. 2014 1 1;14(sup1):S178–S82. 24444203 10.1080/17461391.2012.671368

[4] Enoksen E. Drop-out rate and drop-out reasons among promising Norwegian track and field athletes. Scandinavian sport studies forum. 2011; 2: 19–43.

[5] Møllerløkken NE, Lorås H, Pedersen AV. A systematic review and meta-analysis of dropout rates in youth soccer. Percept Motor Skills. 2015 12 1;121(3):913–922. 10.2466/10.PMS.121c23x0 26595205

[6] Vanden Auweele Y, De Martelaer K, Rzewnicki R, De Knop P, Wylleman P. Parents and coaches: A help or harm? Affective outcomes for children in sport In: Vanden Auweele Y, editor. Ethics in youth sport. Leuven, Belgium: Lannoocampus 2004 p. 72.

[7] Kearney PE, Hayes PR. Excelling at youth level in competitive track and field athletics is not a prerequisite for later success. J Sports Sci. 2018 4 18;36(21):2502–2509. 10.1080/02640414.2018.1465724 29667867

[8] Abbott A, Button C, Pepping GJ, Collins D. Unnatural selection: Talent identification and development in sport. Nonlinear Dynamics Psychol Life Sci. 2005 1;9(1):61–88. 15629068

[9] Den Hartigh RJ, Hill Y, Van Geert PL. The development of talent in sports: A dynamic network approach. Complexity [Internet]. 2018 8 29 [cited 2019 Jul 12] 2018: [about 13 p]. Available from: 10.1155/2018/5094179

[10] Rees T, Hardy L, Güllich A, Abernethy B, Côté J, Woodman T, et al The great British medalists project: A review of current knowledge on the development of the world’s best sporting talent. Sports Med. 2016 8 1;46(8):1041–1058. 10.1007/s40279-016-0476-2 26842017 PMC4963454

[11] Sarmento H, Anguera MT, Pereira A, Araujo D. Talent identification and development in male football: A systematic review. Sports Med. 2018 4 1;48(4):907–931. 10.1007/s40279-017-0851-7 29299878

[12] Ericsson KA, Krampe RT, Tesch-Römer C. The role of deliberate practice in the acquisition of expert performance. Psychol Rev. 1993 6 1;100(3):363–406.

[13] Côté J, Baker J, Abernethy B. Practice and play in the development of sport expertise In: Tenenbaum G, Eklund RC, editors. Handbook of sport psychology. New Jersey: Wiley; 2007 p. 184–202.

[14] Güllich A, Emrich E. Evaluation of the support of young athletes in the elite sports system. Eur J Sport Soc. 2006;3(2):85–108.

[15] Cobley S, Baker J, Wattie N. McKenna J. Annual age-grouping and athlete development. Sports Med. 2009;39(3):235–256. 10.2165/00007256-200939030-00005 19290678

[16] Brazo-Sayavera J, Martínez-Valencia MA, Müller L, Andronikos G, Martindale RJ. Relative age effects in international age group championships: A study of Spanish track and field athletes. PLoS One. 2018 4 24;13(4):e0196386 10.1371/journal.pone.0196386 29689117 PMC5916855

[17] Romann M, Cobley S. Relative age effects in athletic sprinting and corrective adjustments as a solution for their removal. PLoS One. 2015 4 6;10(4):e0122988 10.1371/journal.pone.0122988 25844642 PMC4386815

[18] Carling C, Le Gall F, Reilly T, Williams AM. Do anthropometric and fitness characteristics vary according to birth date distribution in elite youth academy soccer players? Scand J Med Sci Spor. 2009;19(1):3–9. 10.1111/j.1600-0838.2008.00867.x 19000100

[19] Ulbricht A, Fernandez-Fernandez J, Mendez-Villanueva A, Ferrauti A. The relative age effect and physical fitness characteristics in German male tennis players. J Sport Sci Med. 2015;14(3):634–642. PMC4541129 26336351

[20] Helsen WF, Starkes JL, Van Winckel J. The influence of relative age on success and dropout in male soccer players. Am J Hum Biol.1998;10(6):791–8. 10.1002/(SICI)1520-6300(1998)10:6<791::AID-AJHB10>3.0.CO;2-1 28561412

[21] Delorme N, Chalabaev A, Raspaud M. Relative age is associated with sport dropout: Evidence from youth categories of French basketball. Scand J Med Sci Spor. 2011;21(1):120–8. 10.1111/j.1600-0838.2009.01060.x 20136758

[22] Nakata H. Relationship between the relative age effect and lengths of professional careers in male Japanese baseball players: A retrospective analysis. Sports Med Open. 2017;3(1):21–25. 10.1186/s40798-017-0090-3 28577222 PMC5457367

[23] Práxedes A, Moreno A, García-González L, Pizarro D, Del Villar F. The relative age effect on soccer players in formative stages with different sport expertise levels. J Hum Kinet. 2017;60(1):167–173. 29339997 10.1515/hukin-2017-0100 PMC5765797

[24] Ford PR, Ward P, Hodges NJ, Williams AM. The role of deliberate practice and play in career progression in sport: The early engagement hypothesis. High Abil Stud. 2009 6 1;20(1):65–75.

[25] Brazo-Sayavera J, Martínez-Valencia MA, Müller L, Andronikos G, Martindale RJ. Identifying talented track and field athletes: The impact of relative age effect on selection to the Spanish National Athletics Federation training camps. J Sports Sci. 2017 11 23;35(22):2172–8. 10.1080/02640414.2016.1260151 27879175

[26] Hollings SC, Hume PA, Hopkins WG. Relative-age effect on competition outcomes at the World Youth and World Junior Athletics Championships. Eur J Sport Sci. 2014;14(sup1):S456–S61. 24444241 10.1080/17461391.2012.713007

[27] Johnston K, Wattie N, Schorer J, Baker J. Talent identification in sport: A systematic review. Sports Med. 2018;48(1):97–109. 10.1007/s40279-017-0803-2 29082463

[28] Wrang CM, Rossing NN, Diernæs RM, Hansen CG, Dalgaard-Hansen C, Karbing DS. Relative age effect and the re-selection of Danish male handball players for national teams. J Hum Kinet. 2018;63(1):33–41. 30279939 10.2478/hukin-2018-0004 PMC6162975

[29] Gibbs BG, Jarvis JA, Dufur MJ. The rise of the underdog? The relative age effect reversal among Canadian-born NHL hockey players: A reply to Nolan and Howell. Int Rev Sociol Sport. 2012;47(5):644–649.

[30] Côté J, Macdonald DJ, Baker J, Abernethy B. When “where” is more important than “when”: Birthplace and birthdate effects on the achievement of sporting expertise. J Sports Sci. 2006;24(10):1065–1073. 10.1080/02640410500432490 17115521

[31] MacDonald DJ, Cheung M, Côté J, Abernethy B. Place but not date of birth influences the development and emergence of athletic talent in American Football. J Appl Sport Psychol. 2009;21(1):80–90.

[32] Fraser-Thomas J, Côté J, MacDonald DJ. Community size in youth sport settings: Examining developmental assets and sport withdrawal. Revue phénEPS/PHEnex Journal. 2010;2(2). Available from: http://ojs.acadiau.ca/index.php/phenex/article/view/8.

[33] Allen S, Duncan N. Birthplace effect analysis: World Class Programme (WCP) Athletes. London: UK Sport;2010.

[34] MacDonald DJ, King J, Côté J. Abernethy, B. Birthplace effects on the development of female athletic talent. J Sci Med Sport. 2009;12(1):234–237. 10.1016/j.jsams.2007.05.015 17889609

[35] Turnnidge J, Hancock D, Côté J. The influence of birth date and place of development on youth sport participation. Scan J Med Sci Sports. 2014;24(2):461–468. 10.1111/sms.12002 22998526

[36] Elgar FJ, Arlett C, Groves R. Stress, coping, and behavioral problems among rural and urban adolescents. J Adolescence. 2003;26(5):574–585. 10.1016/s0140-1971(03)00057-5 12972270

[37] Hancock DJ, Coutinho P, Côté J, Mesquita I. Influences of population size and density on birthplace effects. J Sports Sci. 2018;36(1):33–38. 10.1080/02640414.2016.1276614 28078945

[38] Kyttä M. Affordances of children's environments in the context of cities, small towns, suburbs and rural villages in Finland and Belarus. J Environ Psychology. 2002;22(1–2):109–123.

[39] Baker J, Logan AJ. Developmental contexts and sporting success: Birth date and birthplace effects in national hockey league draftees 2000–2005. Brit J Sport Med. 2007;41(8):515–517. 10.1136/bjsm.2006.033977 PMC2465449 17331975

[40] Curtis JE, Birch JS. Size of community of origin and recruitment to professional and Olympic Hockey in North America. Sociol Sport J. 1987;4(3):229–44.

[41] Schorer J, Baker J, Lotz S, Büsch D. Influence of early environmental constraints on achievement motivation in talented young handball players. Int J Sport Psychol. 2010;41:42–58.

[42] “Boys and girls champs. regulations and advisory,” Inter-Secondary School Sports Association (ISSA), Jamaica. Available online: https://trackalerts.com/wp-content/uploads/2017/01/Champs-2017-rules.pdf. (accessed on 13 June 2019).

[43] Scott R.A, Irving R, Irwin L, Morrison E, Charlton V, Austin K, et al ACTN3 and ACE genotypes in elite Jamaican and US sprinters. Med Sci Sport Exer. 2010;42(1):107–12. 10.1249/MSS.0b013e3181ae2bc0 20010124

[44] Morrison ES, Cooper P. Some bio-medical mechanisms in athletic prowess. W Indian Med J. 2006;55(3):205–209. 10.1590/s0043-31442006000300015 17087108

[45] Taylor OW. It's culture, not genes: Explaining why Jamaican sprinters are the fastest humans on Earth. Caribbean Quarterly. 2015;61(1):23–41.

[46] Statistical Institute of Jamaica: Population and Housing Census 2011, General Report Volume I, 2012.

[47] Henriksen K, Stambulova N, Roessler KK. Holistic approach to athletic talent development environments: A successful sailing milieu. Psychol Sport Exerc. 2010 5 1;11(3):212–22.

[48] Soberlak P, Cote J. The developmental activities of elite ice hockey players. J Appl Sport Psychol. 2003;15(1):41–49.

[49] Gould D, Horn T. Participation motivation in young athletes In: Silva JM, Weinberg RS, editors. Psychological foundations of sport. Illinois: Human Kinetics; 1984 p. 359–370.

